# Iron Oxide-Cobalt Nanocatalyst for *O*-*tert*-Boc Protection and *O-*Arylation of Phenols

**DOI:** 10.3390/nano8040246

**Published:** 2018-04-17

**Authors:** Vilas B. Gade, Anandarup Goswami, Rajender S. Varma, Sharad N. Shelke, Manoj B. Gawande

**Affiliations:** 1 P. G. & Research Center, Department of Chemistry, S. S. G. M. College, Kopargaon, Dist., Ahmednagar 423601, India; anant.gade1985@gmail.com; 2 Division of Chemistry, Department of Sciences and Humanities, Vignan’s Foundation for Science, Technology and Research Vadlamudi, Guntur 522213, India; 3 Regional Centre of Advanced Technologies and Materials, Faculty of Science, Department of Physical Chemistry, Palacky University, Šlechtitelů 27, 78371 Olomouc, Czech Republic; Varma.Rajender@epa.gov

**Keywords:** magnetic nanocatalysts, Fe_3_O_4_-Co_3_O_4_, *O*-*tert*-butoxycarbonylation, *O*-arylation, phenols, ethers

## Abstract

Efficient and general protocols for the *O*-*tert*-boc protection and *O*-arylation of phenols were developed in this paper using a recyclable magnetic Fe_3_O_4_-Co_3_O_4_ nanocatalyst (Nano-Fe-Co), which is easily accessible via simple wet impregnation techniques in aqueous mediums from inexpensive precursors. The results showed the catalysts were well characterized by XRD (X-ray Diffraction), ICP-AES (Inductive Coupled Plasma Atomic Emission Spectroscopy), TEM (Transmission Electron Microscopy), TOF-SIMS (Time-Of-Flight Secondary Ion Mass Spectrometry) and XPS (X-ray Photoelectron Spectroscopy). The *O*-*tert*-boc protection and *O*-arylation of phenols was accomplished in good to excellent yields (85–95%) and the catalyst was reusable and recyclable with no loss of catalytic activity for at least six repetitions.

## 1. Introduction

Significant research efforts have been devoted to the development of sustainable/greener organic transformations, which are either catalyst-free, solvent-free or performed in an aqueous medium [1,2]. Pollution preventive green and sustainable approaches protect the environment by reducing or eliminating the use of hazardous substances, and avoid by product formation and the generation of unwanted materials. Despite considerable success [3], these protocols, especially those that are catalyst- and solvent-free, are not suitable for all types of reactions and often an efficient and selective catalyst is required to bring out the intended outputs competently. However, in that respect, homogeneous catalysts often face problems of poor stability and recyclability compared to their heterogeneous counterparts. Hence, the design of economical, greener, and recyclable nanocatalysts is highly desirable [4,5,6,7].

In recent years, magnetic supported nanocatalysts have emerged as one of the realistic alternatives to several organic transformations [8,9,10,11,12,13,14], as they are inexpensive, easy to prepare and can be separated (via magnetic decantation) and recycled several times [15]. Using Fe_3_O_4_ as a magnetic support has been popular for the immobilization of diverse metals, namely ruthenium [16,17], palladium [18] and nickel [19]. Such Fe_3_O_4_ supported nanocatalysts are known to catalyze numerous reactions, namely asymmetric Michael additions in aqueous mediums [20]; Suzuki-, Sonogashira-, and Stille-reactions [21]; enantioselective acylation [22]; and Suzuki–Miyaura coupling reactions [23], and has garnered significant attention because of its relevance to industry and academia. However, the full potential of catalytically active species supported by Fe_3_O_4_ for other unattended sustainable organic transformations remains to be explored.

The protection and de-protection of alcohols and phenols is widely applied in industry and academia, especially because of its role in multistep syntheses [24]. However, the success of this method heavily relies on the ease and gentleness of the protection and de-protection protocols. Though, for protection of amino groups, *N*-Boc derivatives are widely employed [24], *O*-Boc protection has started to emerge as an alternative. Nevertheless, most of the synthetic methods used for the synthesis of organic carbonates require the presence of a Lewis base [25] or basic media [26] and the use toxic reagents [27], namely pyridine, phosgene and carbon monoxide. Consequently, efforts have been dedicated to developing eco-friendly protocols for *O*-carbonate synthesis [28,29,30]. In view of the synthetic utility of such protection, specifically regarding the higher stability of the carbonates under basic conditions than the corresponding esters [31], their utility keeps gaining ground, both in academic and industrial research [32]. However, their catalytic versions seem to be relatively unexplored and thus provide us an opportunity to develop sustainable and recyclable nanocatalytic systems for the protection of phenols, in the form of respective carbonates.

The design and synthesis of diaryl ethers is an important strategy in organic chemistry as many naturally occurring compounds comprise of these basic structural units which display activity against several human diseases [33,34]. Numerous catalytic and non-catalytic protocols have been reported for diaryl ether synthesis. For example, the extensive use of Cu, Pd and Ni complexes has been reported for the synthesis of diaryl ethers from aryl halides [35,36,37]. Additionally, CuO nanoparticles were also found to catalyze the *O*-arylation reaction [38]. However, to the best of our knowledge, effective use of heterogeneous nanocatalysts coupled with a greener protocol (e.g., utilization of magnetic support for better separation and recyclability), still remains unexplored for the conversion of phenol derivatives to diaryl ethers.

**Figure 1:**
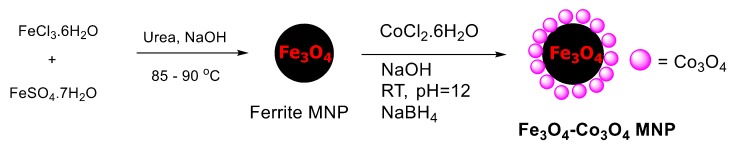
Schematic illustration for the formation of Fe_3_O_4_-Co_3_O_4_MNPs (magnetic nanoparticles).

In continuation of our efforts to develop sustainable, greener, and catalytic organic transformation methods [39,40,41,42,43,44,45,46,47,48,49], in this paper we report an efficient protocol for the *O*-*tert*-butoxycarbonylation and *O*-arylation of phenols using magnetically separable Fe_3_O_4_-Co_3_O_4_MNPs (magnetic nanoparticles), as depicted in Figure 1.

## 2. Results and Discussion

### 2.1. Characterization of the Catalyst

Fe_3_O_4_-Co_3_O_4_ MNPs were prepared by the simple wet impregnation method followed by chemical reduction, as reported in previous literature [47] (Figure 1). Characterization of nanocatalysts was accomplished by transmission electron microscopy (TEM), time-of-flight-secondary ion mass spectrometry (TOF-SIMS), X-ray photoelectron spectroscopy (XPS), X-ray diffraction (XRD), inductive coupled plasma atomic emission spectroscopy (ICP-AES), and SEM elemental mapping with energy dispersive X-ray spectrometry (EDS).

**Figure 2:**
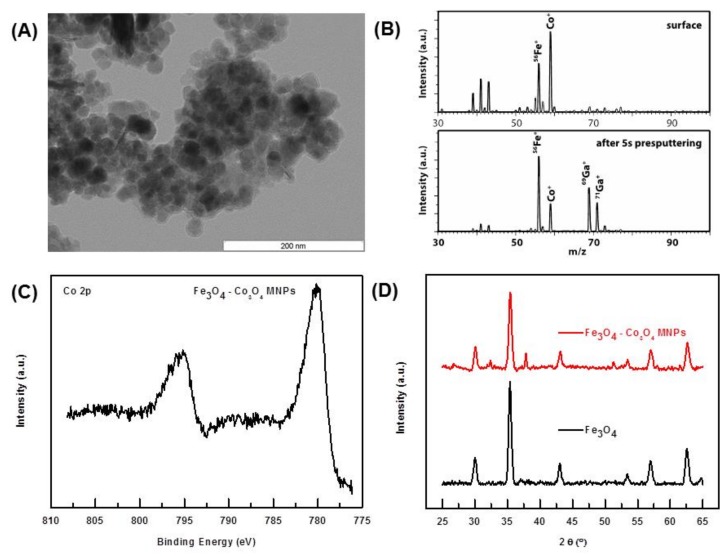
(**A**) TEM image of Fe_3_O_4_-Co_3_O_4_; (**B**) Positive mass spectra of Fe_3_O_4_-Co catalyst: (top) intact surface and (bottom) pre-sputtered surface; (**C**) Co 2p XPS line taken with the energy step of 0.1 eV and acquisition time window of 12 s; (**D**) PXRD spectra of Fe_3_O_4_ (black) and Fe_3_O_4_-Co_3_O_4_.

From TEM images (Figure 2A), the near-spherical shape of the catalysts was confirmed. Based on our previous [48,49] and ongoing works, it can also be extrapolated that Fe_3_O_4_ nanoparticles are surrounded by Co-based nanoparticles on the surface (*vide infra*). The histogram for the delineation of particle size distribution for Fe_3_O_4_-Co_3_O_4_ shows particles to be in the range of 10 nm to 30 nm (Figure S1).

The positive mass spectra of the intact and sputtered surface are shown in Figure 2B. The most intense signal was observed at *m*/*z* 59 followed by the second most intense signal at *m*/*z* 56, corresponding to cobalt (Co^+^) and iron (^56^Fe^+^), respectively. The relative intensities of these two elements affirm that cobalt is mainly on the surface ferrite. We confirmed this from the decrease in relative intensity of the cobalt ion peak with that of iron in a pre-sputtered spectrum, as shown in Figure 2B (bottom).

The oxidation state of Co (and possible connectivity) was established by X-ray Photoelectron Spectroscopy (XPS). The main Co 2p3/2 and Co 2p1/2 peaks, shown in Figure 2C, were at 780.0 eV and 795.6 eV, respectively. Additionally, both peaks were ‘accompanied’ by broad shake-up satellites, meaning that the cobalt was in a paramagnetic state. The position of the Co 2p multiplet, low intensity of the satellites, and their shift with respect to the main peaks of about 8.5 eV, indicate that cobalt was present in the sample as Co_3_O_4_. Indeed, the Co_3_O_4_ spinel surface was characterized by sharp Co 2p peaks at 779.8 eV and 795.7 eV, with the weak and broad satellite structures located about 9 eV higher in binding energy, with respect to the main peaks. However, the presence of Co-Ferrite cannot be completely ruled out [50,51,52,53].

The PXRD spectra of Fe_3_O_4_ and Fe_3_O_4_-Co_3_O_4_ nanoparticles is shown in Figure 2D. The peaks at 30.02, 35.38, 43.02, 53.38, 56.94 and 62.56° 2θ showed the presence of magnetite Fe_3_O_4_ in the sample, and were in good agreement with the reported Fe_3_O_4_ [48]. The small peaks at 32 and 38 belonged to Co_3_O_4_, which was in complete agreement with XPS data [49]. The crystallite size of the Fe_3_O_4_-Co_3_O_4_ MNPs, determined by the Debye Scherrer equation, was found to be 20 nm. Presumably because of the low percentage of Co (7.2% by ICP-AES), the peak for Co was not observable in the XRD spectrum.

**Figure 3:**
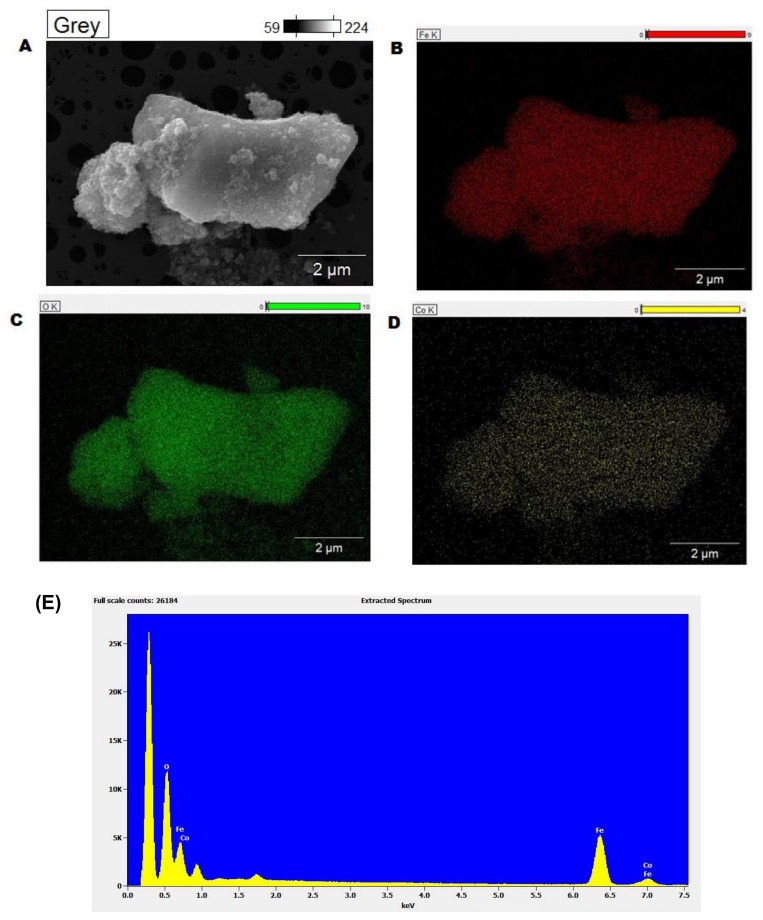
Elemental mapping (**A**–**D**) and energy dispersive X-ray spectrometry (EDX) analysis (**E**) of Fe_3_O_4_-Co_3_O_4_ nanocatalysts.

Elemental mapping of the catalysts clearly showed a homogeneous distribution of iron, cobalt and oxygen on the catalysts (Figure 3A–D). The energy dispersive X-ray spectrometry (EDX) of the sample showed the presence of the same elements. All these characterization data clearly demonstrate the morphology and composition of the nanocatalysts. While Fe_3_O_4_ formed a stable matrix/support for the impregnation of Co-based species, we believe that post-impregnation treatment allowed for the formation of Co_3_O_4_.

### 2.2. Catalytic Applications

The present protocol entailed the treatment of different phenols with Boc (*tert*-butyloxycarbonyl) anhydride ((Boc)_2_O) in presence of Fe_3_O_4_-Co_3_O_4_ MNPs at 70 °C under solvent-free conditions for an appropriate length of time.

**Figure 4:**
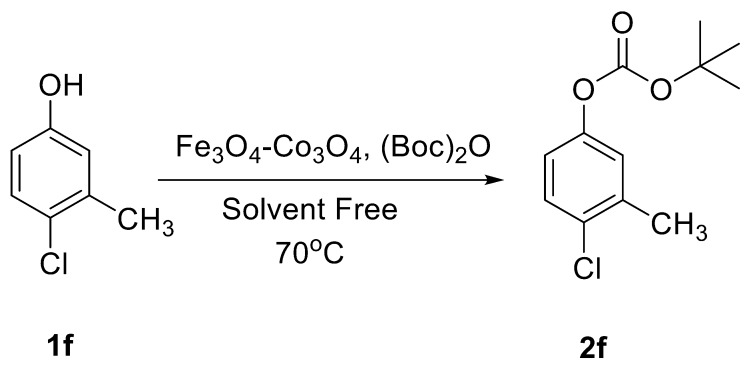
*O*-*tert*-Butoxycarbonylation of 4-chloro-3-methyl phenol.

The catalytic activity of Fe_3_O_4_-Co_3_O_4_ MNPs was explored for the *O*-*tert*-boc protection and the *O*-arylation of phenols. Initially, the optimum reaction conditions were identified for the *O*-*tert*-boc protection of phenols using 4-chloro-3-methyl phenol as a model substrate and Boc anhydride to yield *tert*-Butyl phenyl carbonates (Figure 4). The results are depicted in Table 1.

Initially, the reaction was performed without a catalyst or solvent at RT for 16 h but no product formation was observed (Table 1, entry 1). The same reaction was repeated at 70 °C and trace product formation could be detected after 16 h (Table 1, entry 2). With Fe_3_O_4_-Co_3_O_4_, MNPs (10 mol %) at RT gave 88% of corresponding product, while the same reaction when performed at 70 °C yielded 94% of product (Table 1, entries 3 and 4). Parallel experiments were conducted using 5 mol % of Fe_3_O_4_-Co_3_O_4_ catalysts and bare ferrite MNPs. The results showed 72% and 56% product formation, respectively.

With the optimized conditions, the substrate scope was next explored for the *O*-*tert*-boc protection of phenols (Table 2). The phenols, having electron withdrawing and electron donating groups such as –CH_3_, –Cl, –Br, and –NO_2_, afforded good yields of desired products, including the *O*-*tert*-boc protection of bulky 2-naphthol.

**Figure 5:**
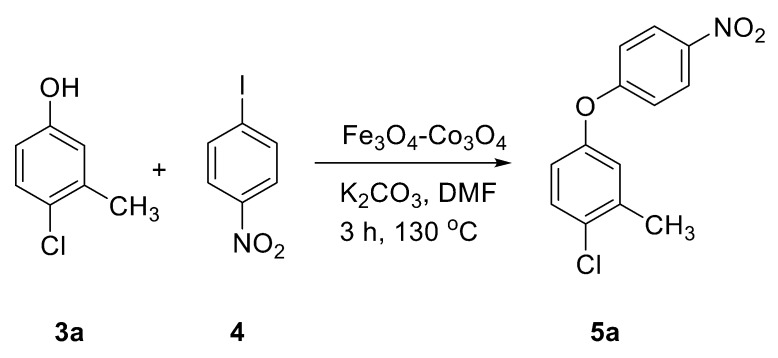
*O*-arylation of 4-chloro-3-methyl phenol.

Catalytic activity was then explored for the *O*-arylation reaction of phenols. At first, the reaction condition was optimized using 4-chloro-3-methyl phenol and 1-iodo-4-nitro benzene as model substrates at 130 °C in the presence of the Fe_3_O_4_-Co_3_O_4_ catalyst (Figure 5). The effects of time, base, solvent and amount of catalyst on progress of reaction were investigated and results are provided in Table 3.

The observed yields were poor in the absence of a catalyst (Table 3, entries 1, 2 and 3), even when different bases were utilized. In the presence of a catalyst, base and with DMF as a solvent, the reaction time was lowered considerably, with significant improvements in yields (Table 3, entries 4 and 5). Parallel experiments with 5 mol % of catalyst and bare ferrite nanoparticles delivered 62% and 58% of corresponding product, respectively (Table 3, entries 6 and 7). After optimizing the reaction conditions, a variety of phenols were subjected to *O*-arylation reactions (Table 4), and good yields (85–94%) of the corresponding products were obtained in most cases. Upon closer inspection of the conversion/yield and the corresponding time needed for each reaction, lower conversion was observed for electron-withdrawing substituents at *p-*position for a given time (e.g., entries 7 and 8). Additionally, an increase in steric bulk (entry 3) also slowed down the reaction rate, leading to less conversion. Nonetheless, the efficiency of the catalytic process, coupled with the relatively clean work-up and recyclability, make this operation economically viable and industrially and academically relevant.

### 2.3. Recycling Study of Fe_3_O_4_-Co_3_O_4_ Nanocatalyst

**Figure 6:**
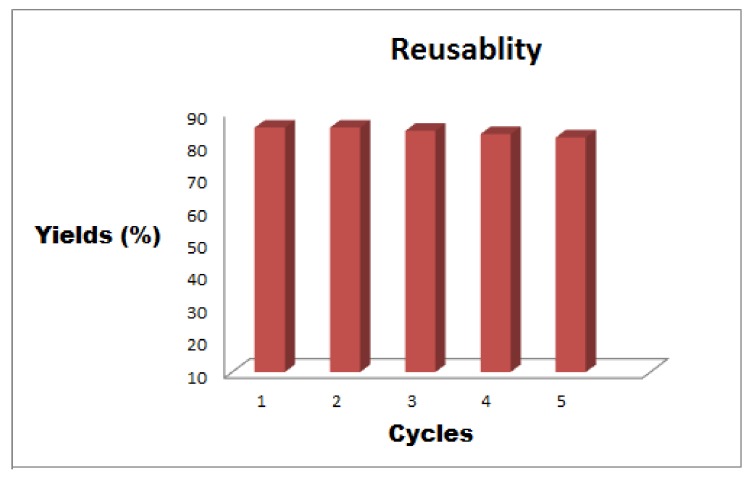
Reusability of Fe_3_O_4_-Co_3_O_4_ MNPs.

The recycling study of the catalyst was performed for *O*-*tert*-butoxycarbonylation of 4-chloro-3-methyl phenol with Boc anhydride using Fe_3_O_4_-Co_3_O_4_ MNPs as a nanocatalyst under optimized reaction conditions. The recycling experiments signified excellent conversions even later than six cycles (Figure 6). After each cycle, the catalyst was magnetically separated, washed with ethyl acetate three times and dried out at 120 °C in oven for 4 h, before being used for the next cycle. After the recyclability study, we did perform XRD of the reused catalyst and noticed that there was no change in the XRD pattern compared to the XRD pattern of the fresh catalyst (Figure S2). The standard deviation of reused catalysts is provided in Table S1.

## 3. Experimental Section

### Materials and Methods

All commercial reagents were used as received without purification. Merck Kieselgel 60 F_254_ precoated aluminium (Merck, Kenilworth, NJ, USA) sheets were used for thin layer chromatography TLC and spots were visualized using iodine and UV light. The IR spectra were scanned on a Perkin Elmer spectrum version 10.4.2 (Perkin Elmer, Waltham, MA, USA). The ^1^H NMR spectra were recorded on a BrukerAvance II 400 (Bruker Company, Billerica, MA, USA) using CDCl_3_ as a solvent. The ^1^H chemical shifts (δ) were reported in ppm relative to internal standard tetramethylsilane. The X-ray powder diffraction pattern was obtained using a conventional powder diffractometer RIGAKU (RIGAKU, Tokyo, Japan), model: MiniFlex™ II benchtop X-ray Diffractometer; and an X-ray tube with Cu-Kα (30 kV/15 mA) radiation operating in Bragg–Brentano (θ/2θ) geometry. Transmission electron microscopy (TEM) experiments were performed on a Hitachi H8100 microscope (Hitachi, Chiyoda, Japan), with a ThermoNoran light element EDS detector and a charged coupled device (CCD) camera for image acquisition. The Fe_3_O_4_-Co fine powder was placed on a carbon stub and the images were recorded at 5–15 kV using a large field detector (LFD) detector under low vacuum. The TOF-SIMS investigations were performed using an upgraded VG Ionex TOF-SIMS instrument (TOF-SIMS IV, Huntingdon, UK) equipped with a Ga^+^ primary ion gun. To obtain the plain surface of the catalysts, the powder sample was pressed on indium before the measurements. Positive and negative secondary ion spectra were collected in the mass range of 0.5–200 *m/z* (T = 5 min) with an upgraded VG Ionex IX23LS TOF-SIMS (Milton Keynes, UK) set-up based on the Poschenrieder design. A focused liquid Ga^+^ gun in pulse mode (6 kHz) was used as a source of analytical ions. The beam current in dc mode at 14 keV was ca. 15 nA with a raster size of 300 × 300 μm^2^. Sample potential was 5 kV. Vacuum during the experiments was maintained in the range of (2–3) × 10^−9^ mbar in the analytical chamber. SEM images were taken using Hitachi SU6600 (Hitachi, Chiyoda, Japan) in the secondary electron mode (SE). The accelerating voltage of 7 kV was used. This microscope was equipped with energy-dispersive spectroscopy (EDS) (Thermo Scientific, Waltham, MA, USA). For EDS mapping, an accelerating voltage of 15 kV and acquisition time of 20,000 s were used.

(a) Preparation of Fe_3_O_4_

The FeCl_3_·6H_2_O (5.4 g) and urea (3.6 g) were dissolved in distilled water (200 mL) at 85–90 °C for 2 h. The brown reaction mixture was cooled to room temperature, to which FeSO_4_·7H_2_O (2.8 g) and then NaOH (0.1 M) was added until the pH reached 10. The molar ratio of Fe^III^ to Fe^II^ was almost 2.00. The ensuing hydroxides were subjected to ultrasonic irradiation in a sealed flask for 30 min at 30 to 35 °C. After aging for 5 h, the ensuing black powder (Fe_3_O_4_) was washed and dried out under vacuum at 60 °C for 24 h.

(b) Preparation of Fe_3_O_4_-Co_3_O_4_MNPs.

Ferrite magnetic nanoparticles Fe_3_O_4_ (2 g) and CoCl_2_·6H_2_O (10 wt % of cobalt on ferrite) were added in water (50 mL) and stirred at room temperature for 1 h. The suspension was adjusted to pH value 12 after impregnation, by adding sodium hydroxide (0.5M) and stirring continuously for 10 to 12 h. The solid was washed with distilled water (5 × 10 mL). The ensuing metal precursors were reduced by adding, dropwise, 0.2 M aqueous NaBH_4_ under mild stirring in an ice-water bath for 30 min until no bubbles were observed in the solution. The resulting Fe_3_O_4_-Co_3_O_4_ MNPs were subjected to ultrasonication for 10 min and then further washed with distilled water and subsequently with ethanol and dried out under vacuum at 60 °C for 24 h.

(c) General Procedure for Boc Protection of Phenols.

Boc_2_O (12 mmol), Fe_3_O_4_-Co_3_O_4_ MNPs (10 mol % with respect to phenol) at room temperature were added to a phenol (10 mmol) and the reaction mixture was stirred at 70 °C under solvent-free conditions for a suitable time. After completion of the reaction (checked by TLC), ethyl acetate (30 mL) was added to the reaction mixture and the catalyst was separated by magnetic decantation. The organic phase was washed with brine solution and subsequently dried on anhydrous sodium sulphate. The crude product was obtained by concentration of organic layers under reduced pressure and was further purified by column chromatography (Silica gel, n-hexane: ethyl acetate) to afford a good yield of the corresponding *O*-*tert*-Boc derivatives.

(d) General Procedure for *O*-arylation Reaction.

A mixture of substituted phenol (10 mmol) and 1-iodo-4-nitro benzene (9 mmol) in DMF (10 mL) was placed in a sealed tube, and potassium carbonate (20 mmol) and Fe_3_O_4_-Co_3_O_4_ catalyst (10 mol % with respect to phenol) was added. The resulting reaction mixture was stirred at 130 °C for the indicated time. After completion of the reaction (as confirmed by TLC) the catalyst was separated by magnetic decantation. The reaction mixture was poured on crushed ice and the contents extracted with ethyl acetate. The combined organic phase was washed with water and brine. It was then dried on anhydrous sodium sulphate and purified by column chromatography (n-hexane: ethyl acetate) to yield a sufficient amount of diaryl ethers.

## 4. Conclusions

In summary, we have developed a recyclable Fe_3_O_4_-Co_3_O_4_ magnetic nanocatalyst expedient for a range of organic transformations, specifically *O*-arylations and the *O*-*tert*-boc protection of phenols. A flexible and active Ferrite-Co catalyst was prepared from inexpensive and abundantly available precursors using simple hydrothermal processes. The Fe_3_O_4_-Co_3_O_4_ nanocatalyst was stable and could be recycled for at least six cycles without significant loss of reactivity. The gentle reaction conditions, easy manipulative procedure, economic viability and proficiency in terms of excellent yields of products, renders this a potentially sustainable option. We believe that the burgeoning field of magnetic nanocatalysts, especially those procured from non-noble metals, has a lot to offer to improve existing organic transformation protocols and the current manuscript exemplified steps to achieve this goal.

## Figures and Tables

**Table 1:** Optimization of *O-tert*-boc protection reaction using 4-chloro-3-methyl phenol and Boc anhydride catalyzed by Fe_3_O_4_-Co_3_O_4_ MNPs ^a^.

No.	Catalyst	Temperature (°C)	Time (h)	Yield ^b^ (2f, %)	TON	TOF (h^−1^)
**1**	--	RT	16	NR	--	--
**2**	--	70	16	Trace	--	--
**3**	Fe_3_O_4_-Co_3_O_4_ MNPs (10 mol %)	RT	16	88	108.3	6.7
**4**	Fe_3_O_4_-Co_3_O_4_ MNPs (10 mol %)	70	3	94	115.7	38.5
**5**	Fe_3_O_4_-Co_3_O_4_ MNPs (5 mol %)	70	3	72	177.3	59.1
**6**	Fe_3_O_4_ MNPs (10 mol %)	70	3	56	5.6	1.8
**7**	Co_3_O_4_NPs	70	3	61	6.1	2

^a^ Reaction conditions: 4-chloro-3-methyl phenol (10 mmol), (Boc)_2_O (12 mmol), catalyst (10 mol % of Co with respect to phenol; the Fe/Co ratio was found to be 1:0.11; NR: No reaction, RT: Room Temperature. ^b^ Isolated Yields.

**Table 2:** Fe_3_O_4_-Co_3_O_4_ catalyzed *O*-*tert*-boc protection of different phenols ^a^.

No.	Phenol	Product	Time (h)	Yield ^b^ (%)
**1**	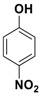 **1a**	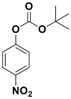 **2a**	2.5	95
**2**	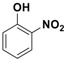 **1b**	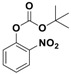 **2b**	3	92
**3**	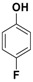 **1c**	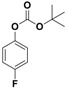 **2c**	3.5	92
**4**	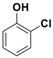 **1d**	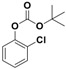 **2d**	4	87
**5**	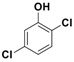 **1e**	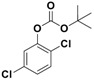 **2e**	3	91
**6**	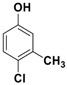 **1f**	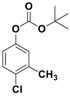 **2f**	3	94
**7**	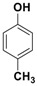 **1g**	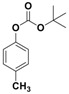 **2g**	3.5	93
**8**	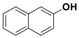 **1h**	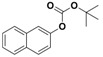 **2h**	4	85

^a^ Reaction conditions: phenol (10 mmol), (Boc)_2_O (12 mmol), catalyst (10 mol % of Co with respect to phenol), 70 °C, ^b^ Isolated Yields.

**Table 3:** Optimization of *O-*arylation reaction using 4-chloro-3-methyl phenol and 1-iodo-4-nitro benzene catalyzed by Fe_3_O_4_-Co_3_O_4_ MNPs ^a^.

No.	Catalyst	Solvent	Base	Time (h)	Yield ^b^ (5a, %)	TON	TOF (h^−1^)
**1**	--	Toluene	K_3_PO_4_	6	10	--	--
**2**	--	DMF	K_3_PO_4_	6	18	--	--
**3**	--	DMF	K_2_CO_3_	6	22	--	--
**4**	Fe_3_O_4_-Co_3_O_4_ (10 mol %)	DMF	K_3_PO_4_	3	72	88.6	29.5
**5**	Fe_3_O_4_-Co_3_O_4_ (10 mol %)	DMF	K_2_CO_3_	3	85	104.6	34.8
**6**	Fe_3_O_4_-Co_3_O_4_ (5 mol %)	DMF	K_2_CO_3_	3	62	152.7	50.9
**7**	Fe_3_O_4_	DMF	K_2_CO_3_	3	58	5.8	1.9
**8**	Co_3_O_4_	DMF	K_2_CO_3_	3	67	6.7	2.2

^a^ Reaction conditions: 4-chloro-3-methyl phenol (10 mmol), 1-iodo-4-nitro benzene (9 mmol), DMF (10 mL), catalyst: 10 mol % of Co with respect to phenol, 130 °C, potassium carbonate (20 mmol), ^b^ Isolated Yields.

**Table 4:** Fe_3_O_4_-Co_3_O_4_ catalyzed *O*-arylation reaction of different phenols ^a^.

No.	Phenol	Product	Time (h)	Yield ^b^ (%)
**1**	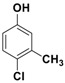 **3a**	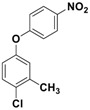 **5a**	3	85
**2**	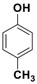 **3b**	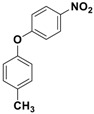 **5b**	3	89
**3**	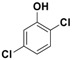 **3c**	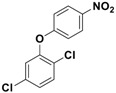 **5c**	4	91
**4**	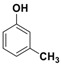 **3d**	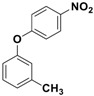 **5d**	3.5	87
**5**	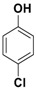 **3e**	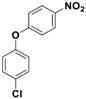 **5e**	3	88
**6**	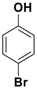 **3f**	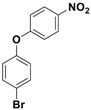 **5f**	3	91
**7**	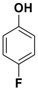 **3g**	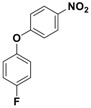 **5g**	3.5	94
**8**	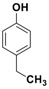 **3h**	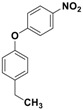 **5h**	3	90

^a^ Reaction conditions: phenol (10 mmol), 1-iodo-4-nitro benzene (9 mmol), DMF (10 mL), catalyst: 10 mol % of Co with respect to phenol, 130 °C, potassium carbonate (20 mmol), ^b^ Isolated Yields.

## Supplementary Materials

The following are available online at http://www.mdpi.com/2079-4991/8/4/246/s1. Figure S1: Calculation of standard deviation, Figure S2: NMR spectra of synthesized compound.
