# Supplementary material for: Iron Oxide-Cobalt Nanocatalyst for O-tert-Boc Protection and O-Arylation of Phenols
Source: Nanomaterials (Basel). 2018 Apr 17;8(4):246. doi: 10.3390/nano8040246 (PMC5923576; doi:10.3390/nano8040246)
Supplement: Supplementary file 1 [file nanomaterials-08-00246-s001.pdf]

nanomaterials

Article

Iron oxide-Cobalt Nanocatalyst for *O*-*tert*-Boc Protection and *O*-Arylation of Phenols

Vilas B. Gade[1],AnandarupGoswami[2,*], Rajender S. Varma[3], Sharad N. Shelke[1,*] and Manoj B. Gawande [3,*]

[1] P. G. & Research Center, Department of Chemistry, S. S. G. M. College, Kopargaon, Dist., Ahmednagar 423601, India; anant.gade1985@gmail.com
[2] Division of Chemistry, Department of Sciences and Humanities, Vignan's Foundation for Science, Technology and Research Vadlamudi, Guntur 522213, India
[3] Regional Centre of Advanced Technologies and Materials, Faculty of Science, Department of Physical Chemistry, Palacky University, Šlechtitelů 27, 78371 Olomouc, Czech Republic; Varma.Rajender@epa.gov
*Correspondence: ananda1911@gmail.com (A.G.); snshelke@yahoo.co.in (S.N.S.); manoj.gawande@upol.cz (M.B.G.); Tel.: +420-58-563-4544 (M.B.G.)

Calculations for the size of nanoparticles

In order to build the histogram for the particle size distribution of Fe_3O_4-Co_3O_4, a large number of images (10 to 15) were acquired from each sample (for example, one representative image is shown below Figure S1) and the size of the particles on the border area was measured. It was noted that most of the particles are in the range from 10-30 nm; though the presence of small size nanoparticles cannot be excluded.

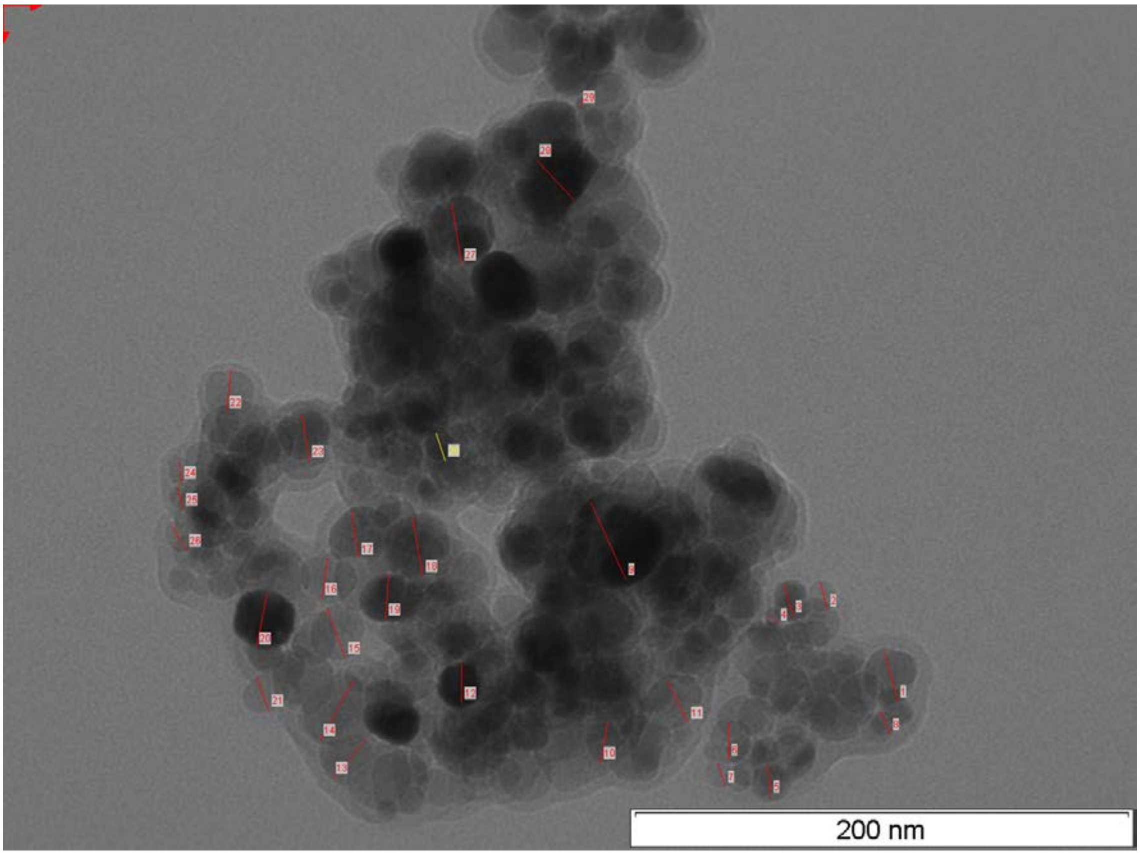

Figure S1. Representative Fe_3O_4-Co_3O_4 TEM image for histogram calculation.

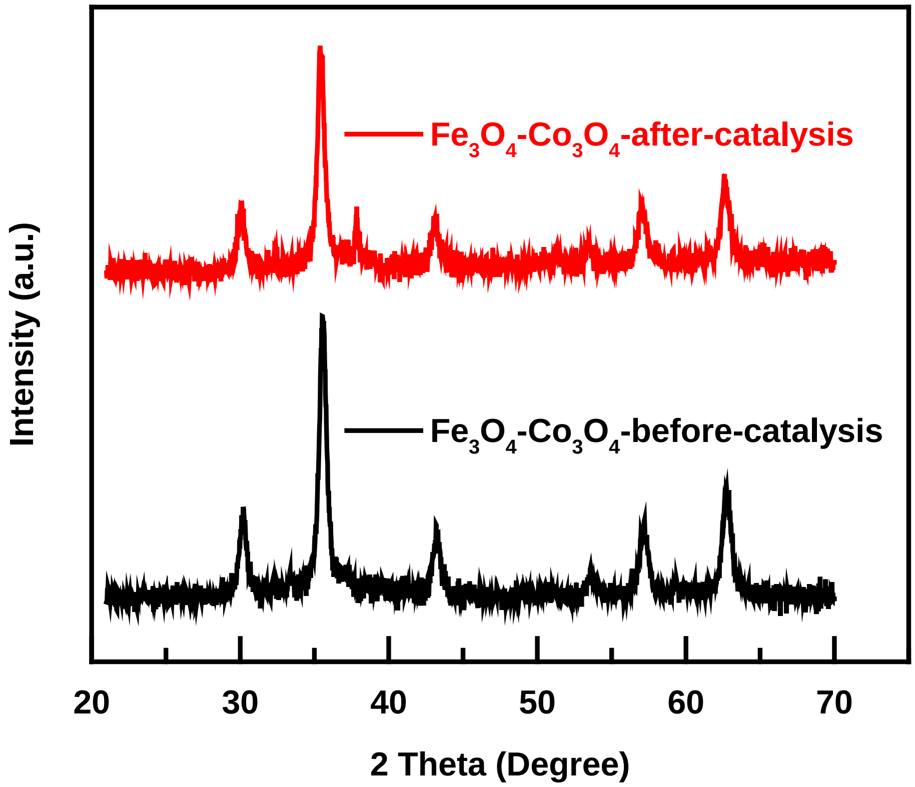

Figure S2. XRD spectra of fresh and reused catalyst.

Standard deviation of catalytic test

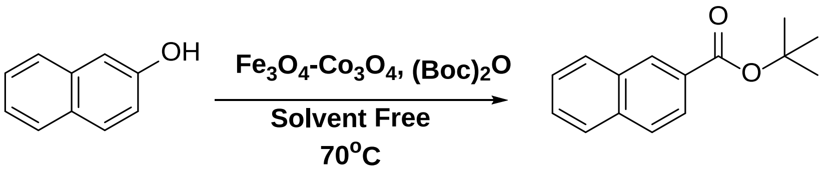

1h **2h**

Run	Yield (%)	Standard Deviation (%)
1	85	2.14
2	85	
3	84	
4	82	
5	81	
6	80	

S.D. = $\sqrt{\sum |x-\mu|^2 / N-1}$

X = individual value

μ = mean

N = Number of measurements

μ = 82.83

$\sum |x-\mu|^2$ = 22.8334

N = 6

The comparison of 0.250 g of Fe_3O_4-Co_3O_4 nanocatalyst taken 6 times in parallel for O-*tert*-butoxycarbonylation of 2-naphthol.

Number	Mass of Fe_3O_4-Co_3O_4 Nanocatalysts (mg)[a]	Standard Deviation (mg)
1	250	2.07
2	249	
3	245	
4	245	
5	248	
6	247	

[a]Mass of catalyst after magnetic cleansing, washing and vacuum drying

S.D. = $\sqrt{\sum |x-\mu|^2 / N-1}$

X = individual value

μ = mean

N = Number of measurements

μ = 247.33

$\sum |x-\mu|^2$ = 21.3334

N = 6

Spectral Data

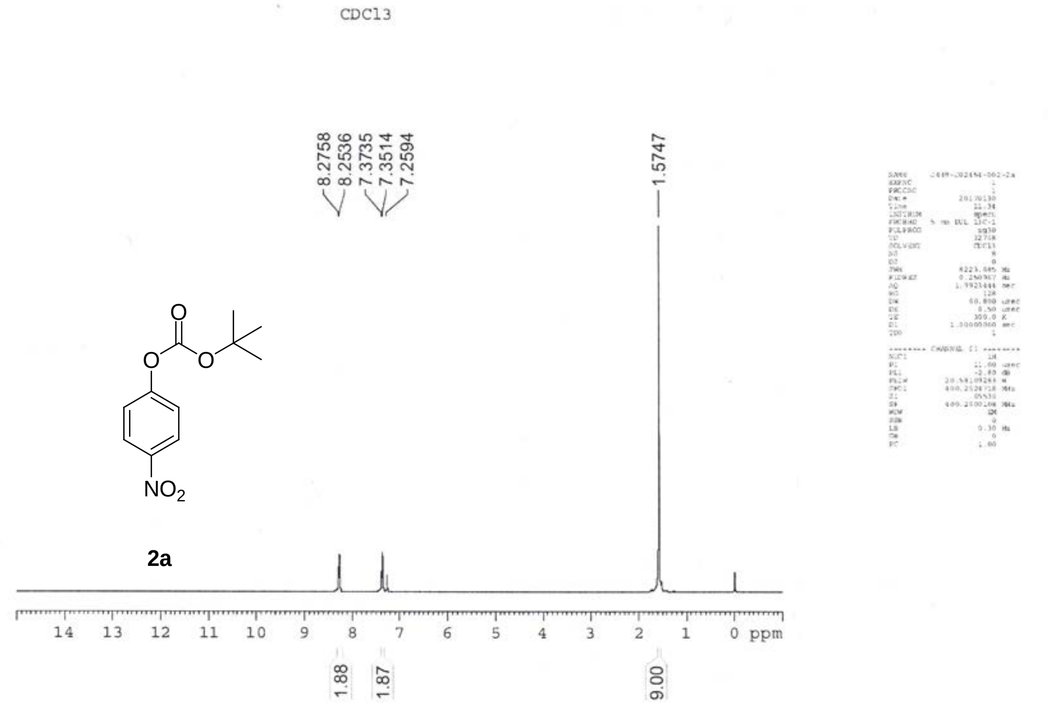

2a. IR (ν, cm^{-1}): 2987.5, 1753.42, 1614.86, 1521.63, 1344.97, ^{1}H NMR (400 MHz, $CDCl_3$, δ, ppm): 1.57 (9H, s, CH_3), 7.35-7.37 (2H, d, *J* = 8.8 Hz, HAr), 8.25-8.27 (2H, d, *J* = 8.8 Hz, HAr), LC-MS: 239 [M^{+}]

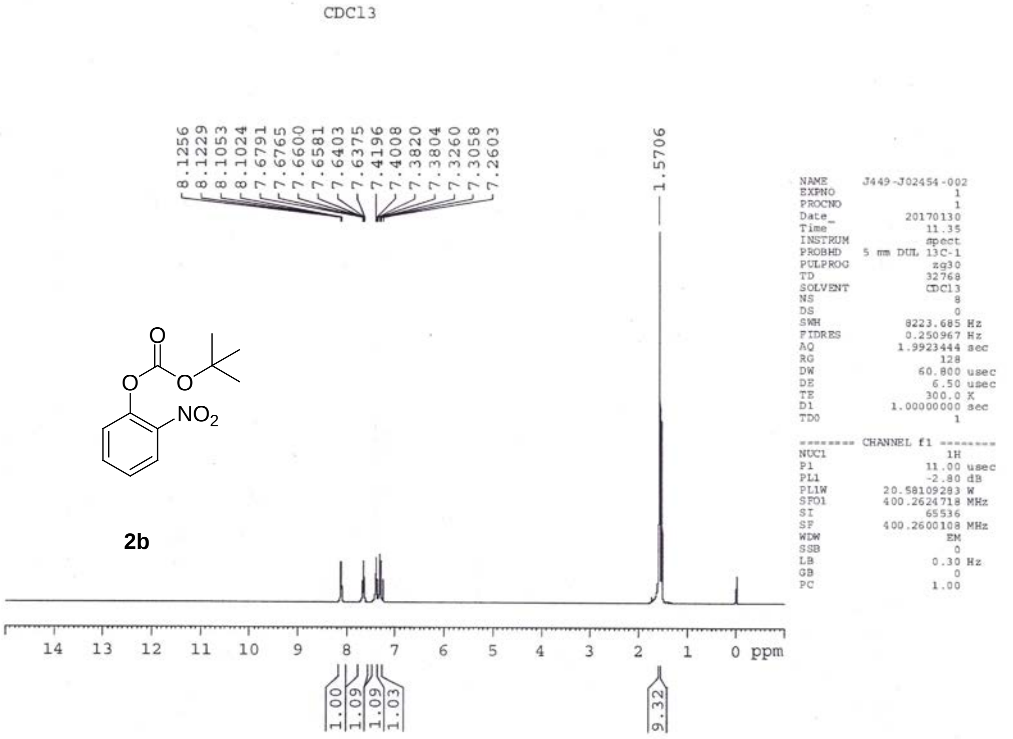

2b. IR (ν, cm^{-1}): 2983.85, 1763.32, 1605.32, 1530.09, 1328.99, ^{1}HNMR (400 MHz, $CDCl_3$, δ, ppm): 1.57 (9H, s, CH_3), 7.30-7.32 (1H, d, *J* = 8.1 Hz, HAr), 7.38-7.41 (1H, t, *J* = 7.5 Hz, HAr), 7.63-7.67 (1H, d, *J* = 1 & 7. 6 Hz, HAr), 8.10-8.12 (1H, dd, *J* = 1 & 8.1 Hz, HAr), LC-MS: 239 [M+1]$^{+}$

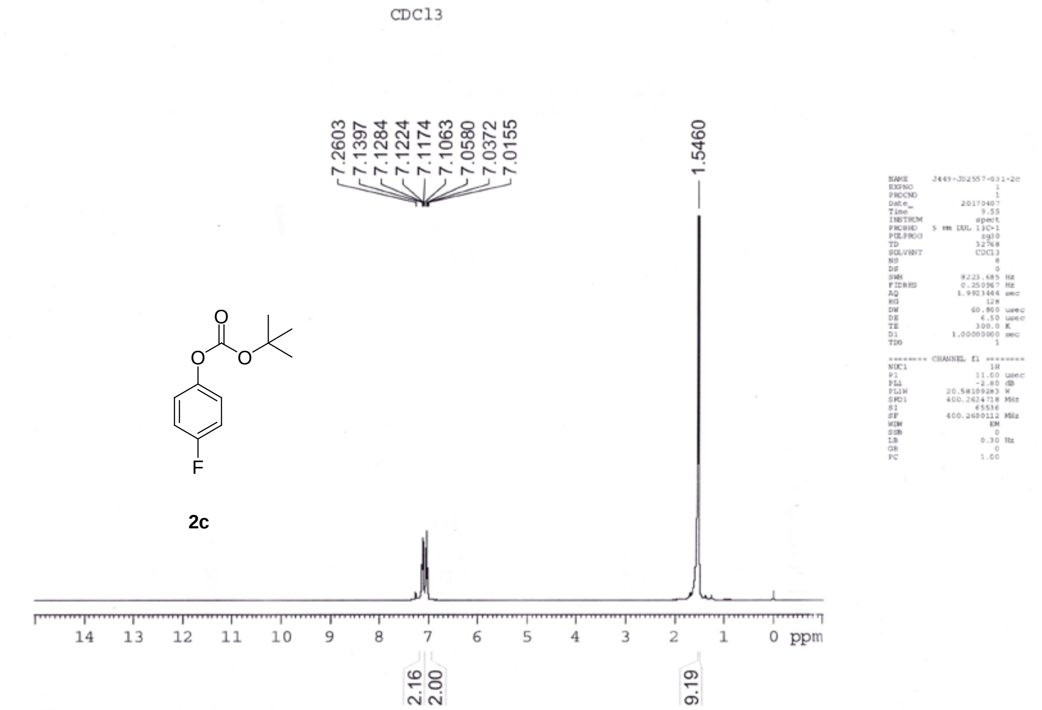

2c.IR (ν, cm^{-1}): 2982.69, 1756.22, 1605, 1505.70, 1371.29, 1141.99, ^{1}HNMR (400 MHz, $CDCl_3$, δ, ppm): 1.54 (9H, s, CH_3), 7.01-7.05 (2H, m, HAr), 7.10-7.13 (2H, m, HAr), LC-MS: 212 [M+Na]$^+$

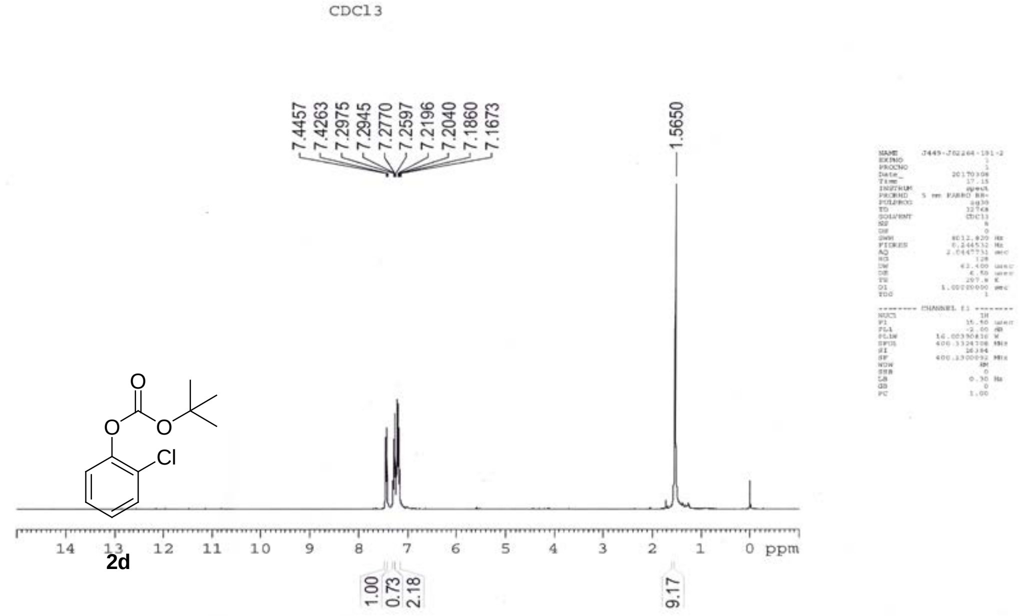

2d. IR (ν, cm^{-1}): 2982.33, 1761.02, 1605, 1251.52, 1062.67, ^{1}HNMR (400 MHz, $CDCl_3$, δ, ppm): 1.56 (9H, s, CH_3), 7.16-7.21 (2H, m, HAr), 7.25-7.29 (1H, m, HAr), 7.42-7.44 (1H, d, *J* = 7.7 Hz, HAr) LC-MS: 228 [M+18]$^+$

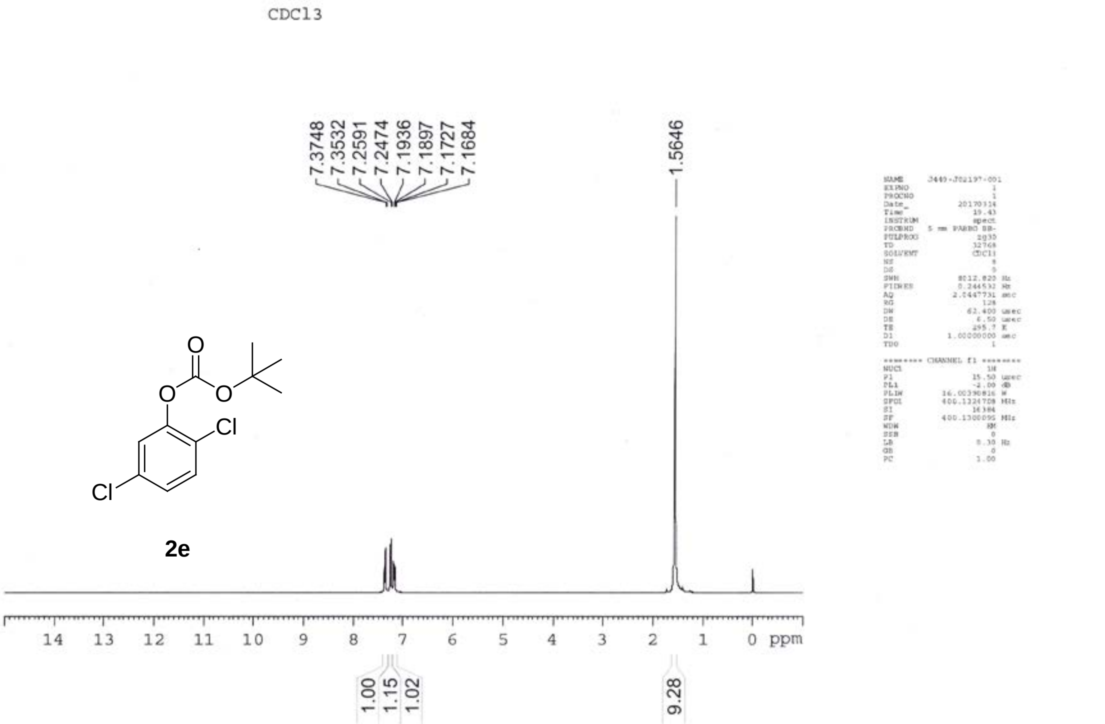

2e. IR (ν, cm^{-1}): 2982.99, 1762.93, 1576.70, 1252.17, 1060.45, ^{1}HNMR (400 MHz, $CDCl_3$, δ, ppm): 1.56 (9H, s, CH_3), 7.16-7.19 (1H, dd, *J* = 1.5 & 8.6 Hz HAr), 7.24 (1H, s, HAr), 7.35-7.37 (1H, d, *J* = 8.6 Hz, HAr), LC-MS: 262 $[M+2]^+$

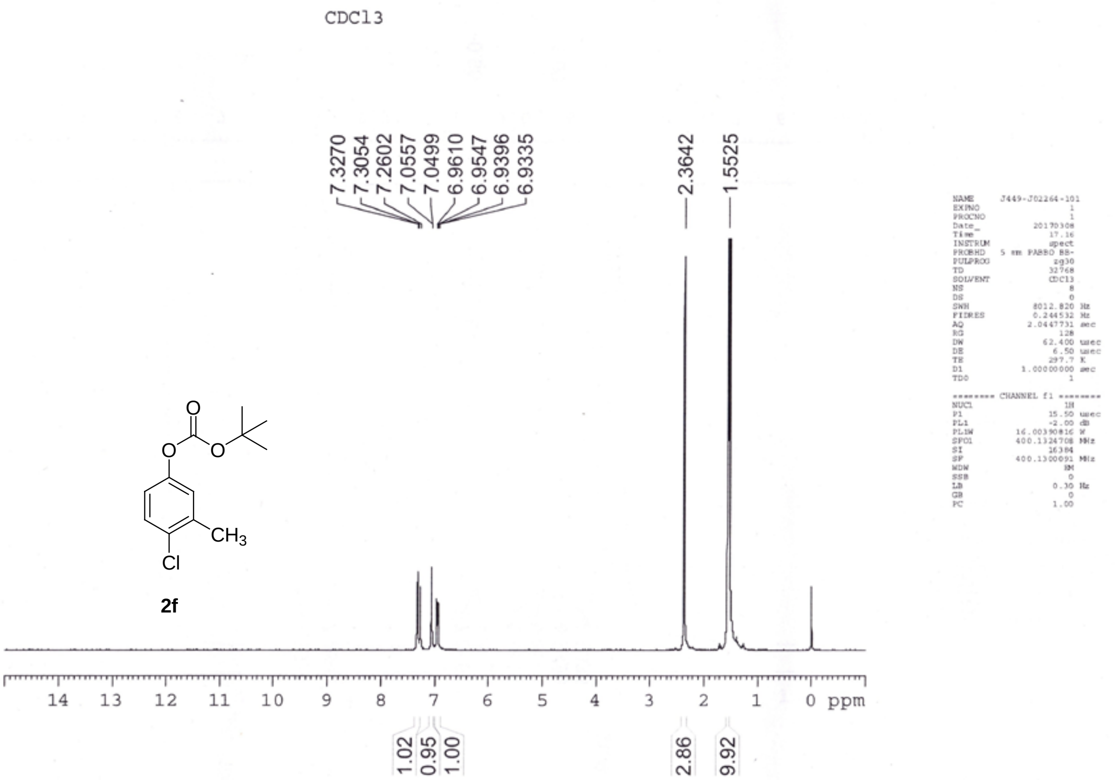

2f. IR (ν, cm^{-1}): 2982.35, 1756.18, 1602, 1250.36, 1060.09, ^{1}HNMR (400 MHz, $CDCl_3$, δ, ppm): 1.55 (9H, s, CH_3), 2.36 (3H, s, CH_3), 6.93-6.96 (1H, dd, *J*= 8.5 & 2.5 Hz HAr), 7.04-7.05 (1H, d, *J*=2.3 Hz, HAr), 7.30-7.32 (1H, d, *J*=8.6 Hz, HAr), LC-MS: 242 [M+18]$^+$

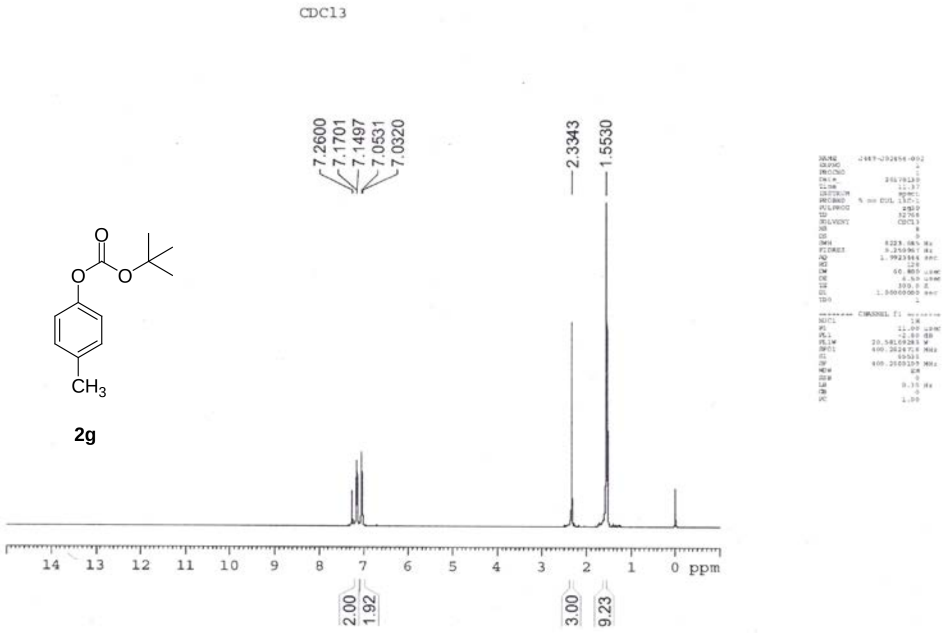

2g. IR (ν, cm^{-1}): 2981.75, 1754.38, 1602, 1254.62, ^{1}HNMR (400 MHz, $CDCl_3$, δ, ppm): 1.55 (9H, s, CH_3), 2.33 (3H, s, CH_3), 7.03-7.05 (2H, d, *J*= 8.4 Hz, HAr), 7.14-7.17 (2H, d, *J*= 8.2 Hz, HAr), LC-MS: 208 [M+18]$^+$

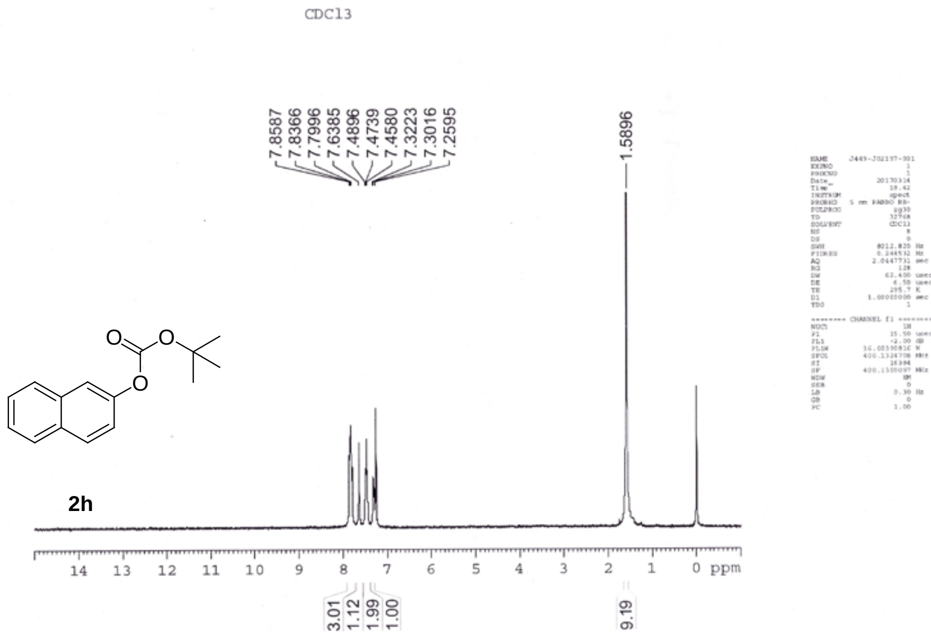

2h. IR (ν, cm^{-1}): 2982.67, 1746.56, 1599.42, 1236.35, ^{1}HNMR (400 MHz, $CDCl_3$, δ, ppm): 1.58 (9H, s, CH_3), 7.30-7.32 (2H, d, *J*= 8.2 Hz, HAr), 7.45-7.48 (2H, m, HAr), 7.63 (1H, S, HAr), 7.79-7.85 (3H, m, HAr), LC-MS: 244 [M+18]$^+$

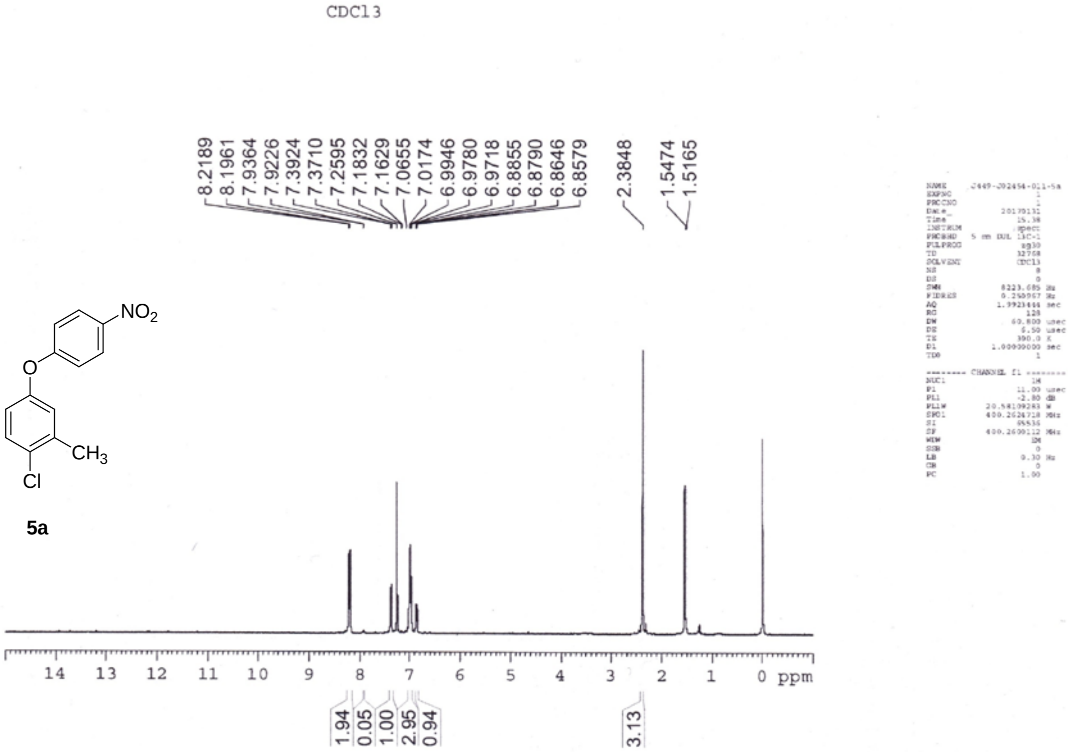

5a. IR (ν, cm^{-1}): 2925.7, 1574.68, 1506.98, 1339.86, 1240.61, ^{1}HNMR (400 MHz, $CDCl_3$, δ, ppm): 2.38 (3H, s, CH_3), 6.85-6.88 (1H, dd, *J* = 2.6 & 8.3 Hz, HAr), 6.97-6.98 (1H, d, *J* =2.4 Hz, HAr), 6.99-7.01 (2H, d, *J* = 9.1 Hz, HAr), 7.37-7.39 (1H, d, *J* = 8.5 Hz, HAr), 8.19-8.21 (2H, d, *J* =9.1 Hz, HAr), LC-MS: 263[M+1]$^+$

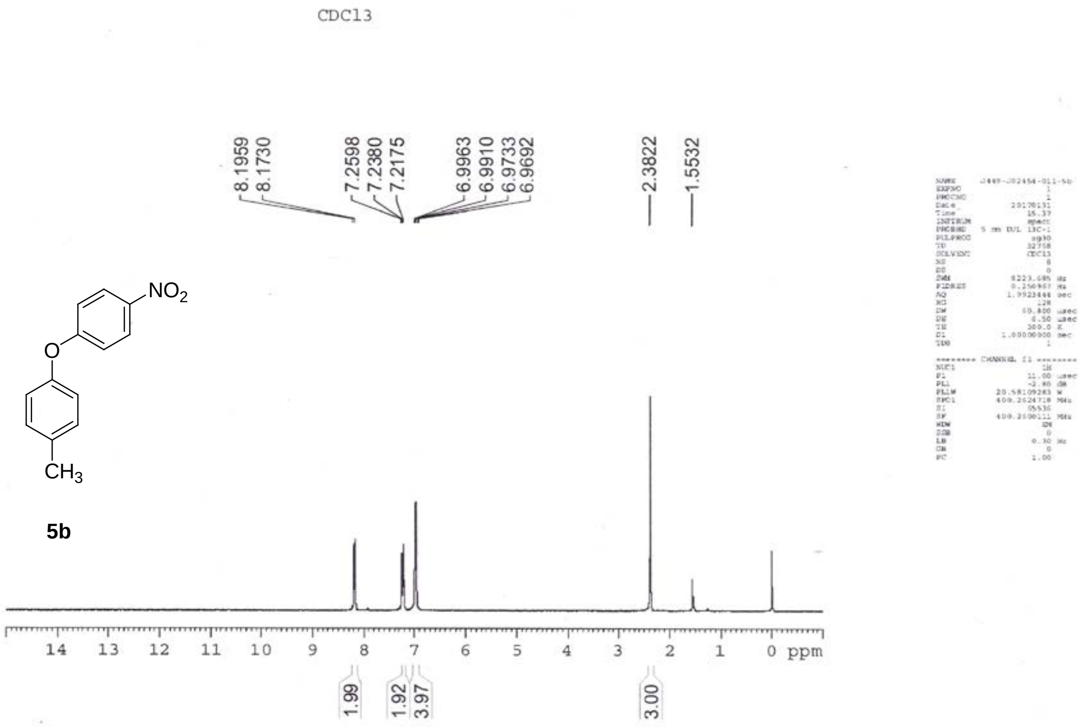

5b. IR (ν, cm^{-1}): 2924, 1587.80, 1505.09, 1338.20, 1243.33, ^{1}HNMR (400 MHz, $CDCl_3$, δ, ppm): 2.38 (3H, s, CH_3), 6.96-6.99 (4H, d, HAr), 7.21-7.23 (2H, d, *J*= 8.2 Hz, HAr), 8.17-8.19 (2H, d, *J* = 9.1 Hz, HAr), LC-MS: 229 [M-4]$^+$

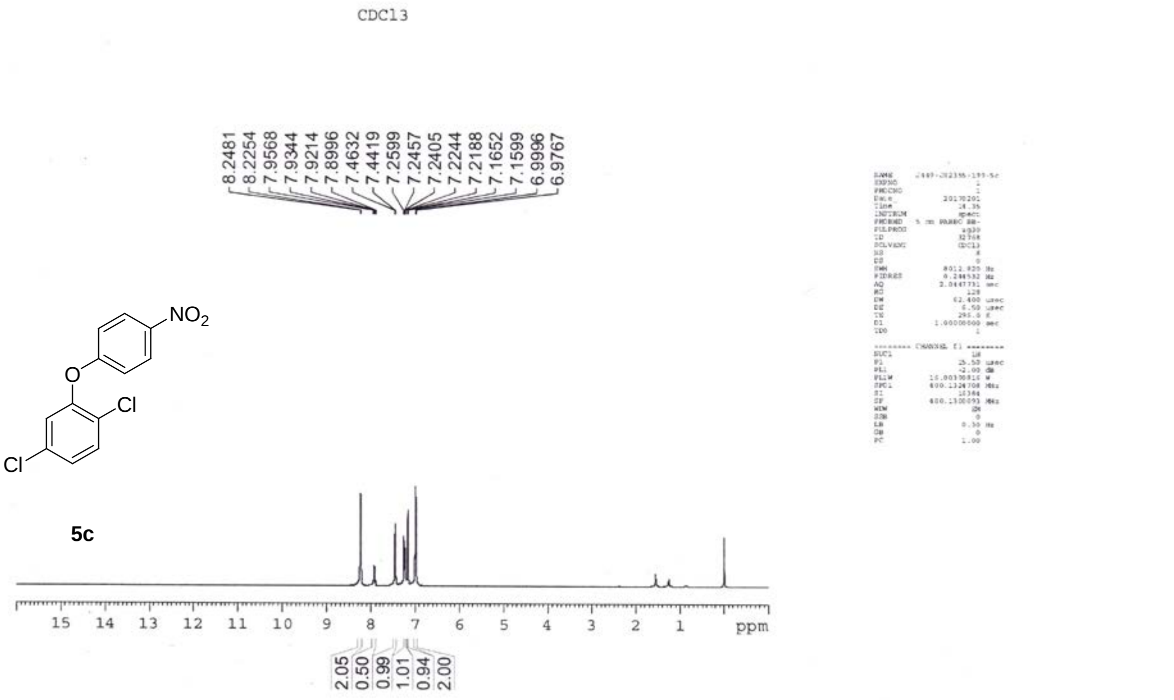

5c. IR (ν, cm^{-1}): 2926.7, 1568.86, 1509.75, 1339.51, 1235.56, 1083.11, ^{1}HNMR (400 MHz, $CDCl_3$, δ, ppm): 6.97-6.99 (2H, d, *J* = 9.1 Hz, HAr), 7.15-7.16 (1H, d, *J* = 2.1 Hz, HAr), 7.21-7.24 (1H, dd, *J* = 2.2 & 8.7 Hz, HAr), 7.44-7.46 (1H, d, *J* = 8.5 Hz, HAr), 8.22-8.24 (2H, d, *J* = 9.1 Hz, HAr), LC-MS: 284 $[M]^+$

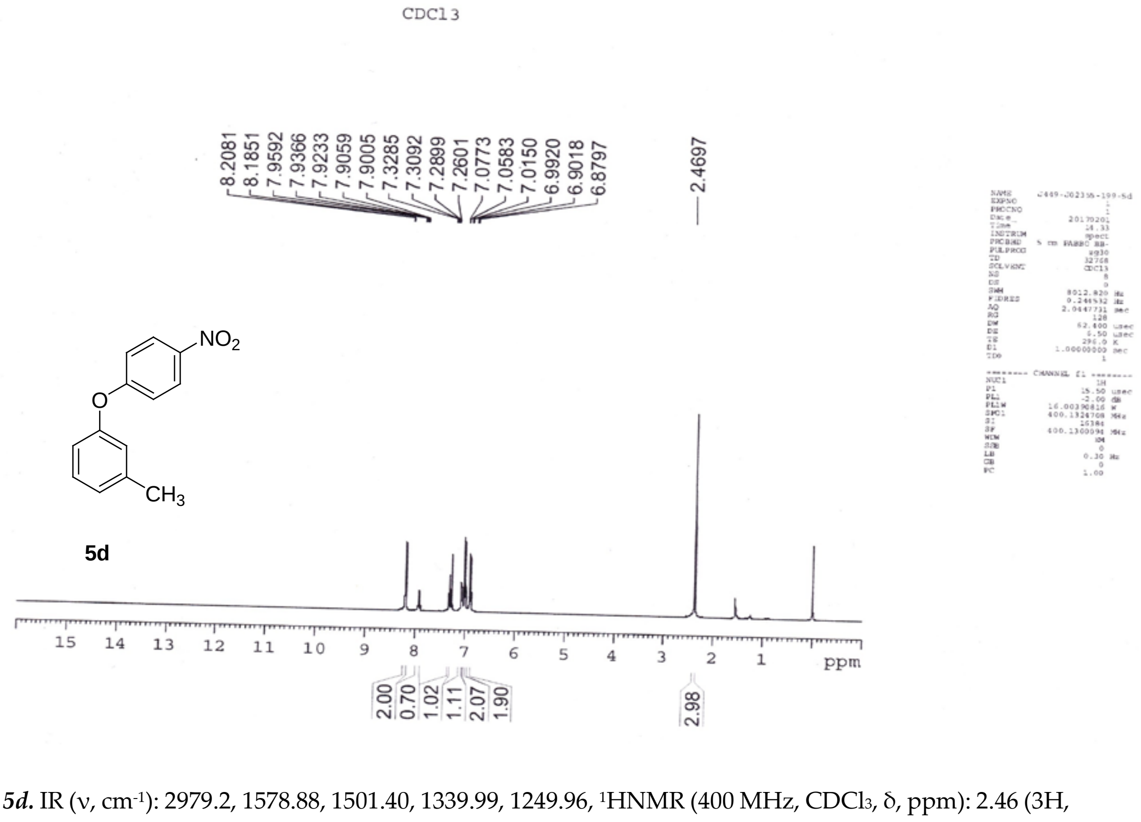

5d. IR (ν, cm^{-1}): 2979.2, 1578.88, 1501.40, 1339.99, 1249.96, ^{1}HNMR (400 MHz, $CDCl_3$, δ, ppm): 2.46 (3H, s, CH_3), 6.87-6.90 (2H, m, HAr), 6.99-7.01 (2H, d, *J*= 9.2 Hz, HAr), 7.05-7.07 (1H, d, *J*= 7.6 Hz, HAr), 7.28-7.32 (1H, t, *J*= 7.7 Hz, HAr), 8.18-8.20 (2H, d, *J*= 9.2 Hz, HAr), LC-MS: 229 $[M+1]^+$

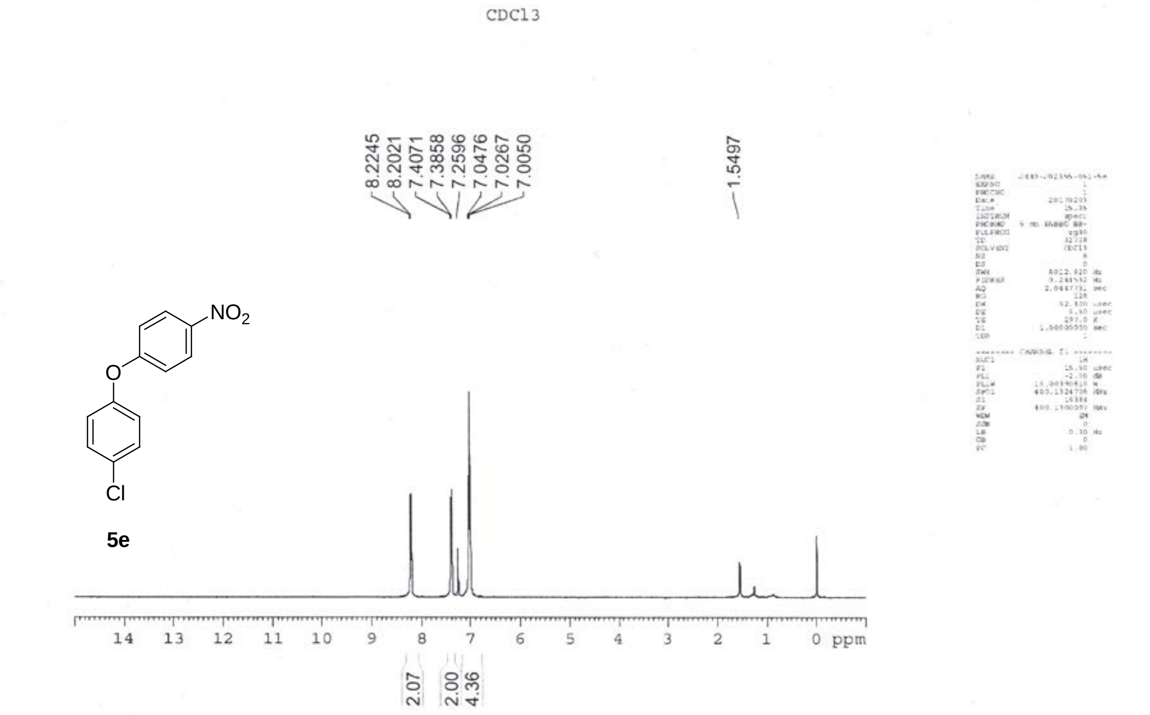

***5e*.** IR (ν, cm[-1]): 2924, 1610.2, 1598.5, 1510.59, 1340.54, 1244.06, 1088.74, ^{1}HNMR (400 MHz, $CDCl_3$, δ, ppm): 7.00-7.04 (4H, m, HAr), 7.38-7.40 (2H, d, *J* = 8.5 Hz, HAr), 8.20-8.22 (2H, d, *J*= 8.9 Hz, HAr), LC-MS: 249, 250 $[M+1]^+$, $[M+2]^+$

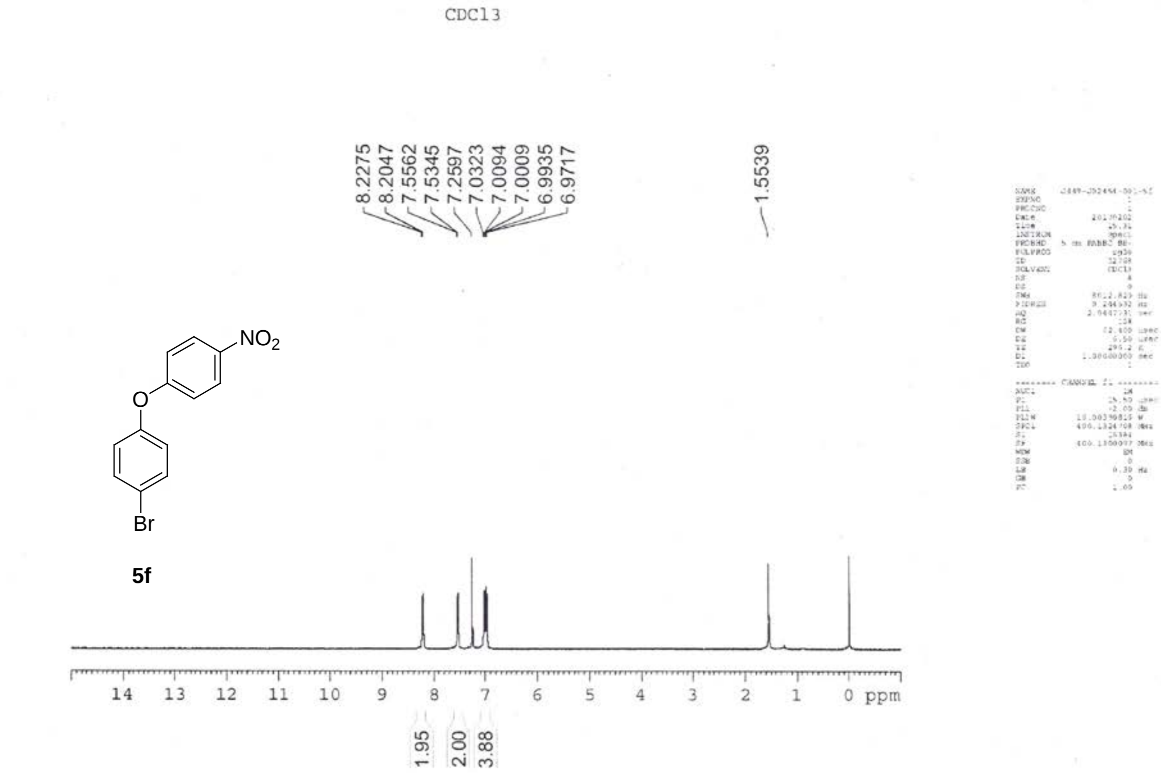

***5f*.** IR (ν, cm[-1]): 2965.4, 1610, 1509.2, 1339.67, 1241.97, 843.25, ^{1}HNMR (400 MHz, $CDCl_3$, δ, ppm): 6.97-6.99 (2H, d,*J* = 8.7 Hz, HAr), 7.00-7.03 (2H, d, *J* = 9.1 Hz, HAr), 7.53-7.55 (2H, d, *J* = 8.7 Hz, HAr), 8.20-8.22 (2H, d, *J* = 9.1 Hz, HAr), LC-MS: 294 $[M]^+$

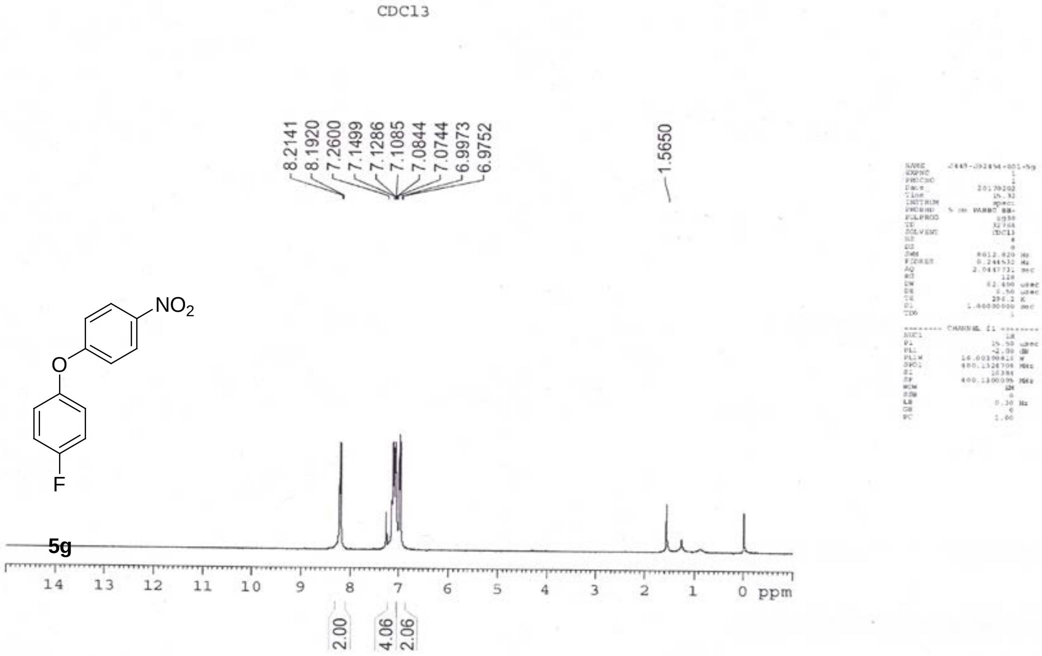

5g. IR (ν, cm^{-1}): 2924.62, 1602.71, 1500.65, 1336.94, 1185.39, 1215.03, ^{1}HNMR (400 MHz, $CDCl_3$, δ, ppm): 6.97-6.99 (2H, d, *J* = 8.8 Hz, HAr), 7.07-7.14 (4H, m, HAr), 8.19-8.21 (2H, d, *J* = 8.8 Hz, HAr), LC-MS: 233 [M-1]$^{+}$

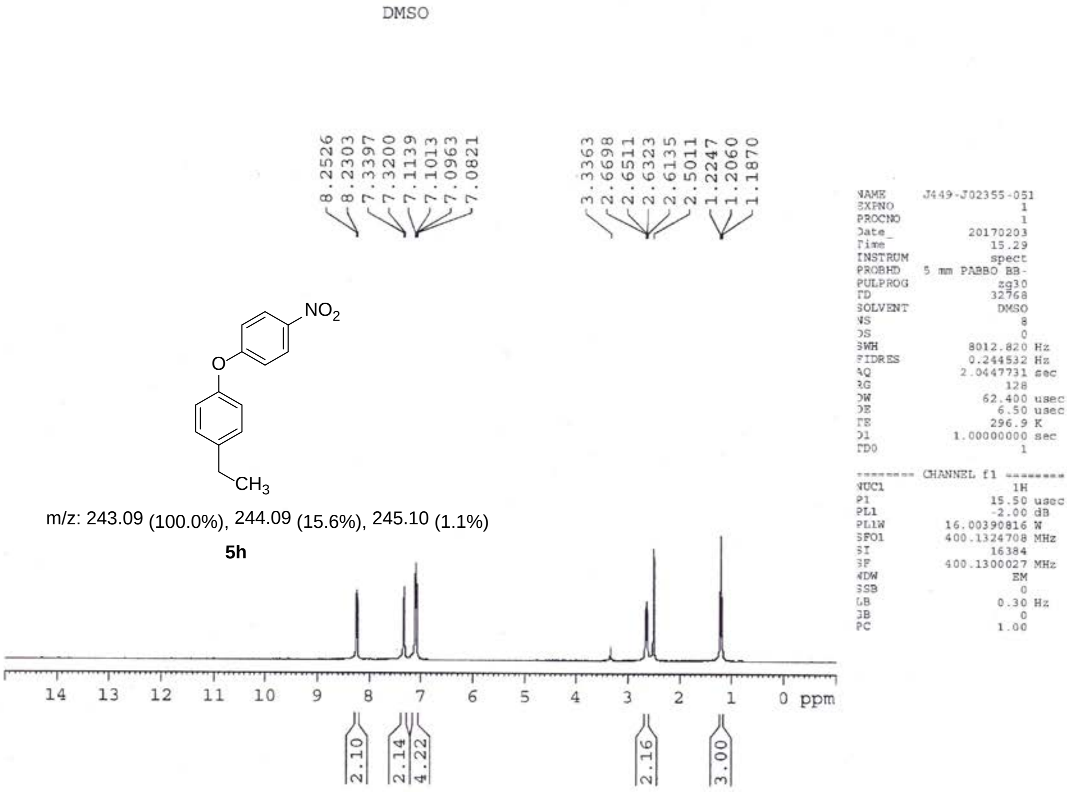

5h. IR (ν, cm^{-1}): 2966.09, 1609.13, 1587.23, 1505. 63, 1340.17, 1242.03, ^{1}HNMR (400 MHz, DMSO-d_6, δ, ppm): 1.18-1.22 (3H, t, *J* = 7.6 Hz, CH_3), 2.61-2.66 (2H, q, *J* = 7.5 Hz, CH_2), 7.08-7.11 (4H, m, HAr), 7.32-7.33 (2H, d, *J* = 7.9 Hz, HAr), 8.23-8.25 (2H, d, *J* = 8.9 Hz, HAr), LC-MS: 243 [M-1]$^{+}$
